# Comparison of the medical students’ perceived self-efficacy and the evaluation of the observers and patients

**DOI:** 10.1186/1472-6920-13-49

**Published:** 2013-04-08

**Authors:** Jette Ammentorp, Janus Laust Thomsen, Dorte Ejg Jarbøl, René Holst, Anne Lindebo Holm Øvrehus, Poul-Erik Kofoed

**Affiliations:** 1 Health Services Research Unit, Lillebaelt Hospital/IRS University of Southern Denmark, Vejle, Denmark; 2 Research Unit of General Practice, Institute of Public Health, University of Southern Denmark, Odense, Denmark; 3 Department of Biostatistics, Institute of Regional Health Service Research University of Southern Denmark, Odense, Denmark; 4 Education Development Unit, Faculty of Health Sciences, University of Southern Denmark, Odense, Denmark; 5 Department of Paediatrics, Kolding Hospital, Kolding, Denmark

**Keywords:** Self-assessment, Self-efficacy, Calgary Cambridge observation guide, Communication skills training

## Abstract

**Background:**

The accuracy of self-assessment has been questioned in studies comparing physicians’ self-assessments to observed assessments; however, none of these studies used self-efficacy as a method for self-assessment.

The aim of the study was to investigate how medical students’ perceived self-efficacy of specific communication skills corresponds to the evaluation of simulated patients and observers.

**Methods:**

All of the medical students who signed up for an Objective Structured Clinical Examination (OSCE) were included. As a part of the OSCE, the student performance in the “parent-physician interaction” was evaluated by a simulated patient and an observer at one of the stations. After the examination the students were asked to assess their self-efficacy according to the same specific communication skills.

The Calgary Cambridge Observation Guide formed the basis for the outcome measures used in the questionnaires.

A total of 12 items was rated on a Likert scale from 1–5 (strongly disagree to strongly agree).

We used extended Rasch models for comparisons between the groups of responses of the questionnaires. Comparisons of groups were conducted on dichotomized responses.

**Results:**

Eighty-four students participated in the examination, 87% (73/84) of whom responded to the questionnaire. The response rate for the simulated patients and the observers was 100%.

Significantly more items were scored in the highest categories (4 and 5) by the observers and simulated patients compared to the students (observers versus students: -0.23; SE:0.112; p=0.002 and patients versus students:0.177; SE:0.109; p=0.037). When analysing the items individually, a statistically significant difference only existed for two items.

**Conclusion:**

This study showed that students scored their communication skills lower compared to observers or simulated patients. The differences were driven by only 2 of 12 items.

The results in this study indicate that self-efficacy based on the Calgary Cambridge Observation guide seems to be a reliable tool.

## Background

Communication skills training has increasingly become a part of the training of healthcare professionals and it appears to be evident and generally accepted that communication skills are core competencies essential for good patient care [1].

Different methods for teaching and assessing communication skills training have been investigated [2-5]. The majority of the studies have insufficient information about the communication behaviour taught, and in many studies a mismatch exists between the stated behaviour and the assessment instrument used [5]. Clearly, assessment instruments closely matching the communication skills taught should be used to gain a clear sense of the impact of the training assessed [5]. As opposed to self-assessment, external assessment uses objective measures rated by an examiner and has mainly been used for evaluating performance during exam situations. Self-assessment is used to assess the outcome of continuous professional development using questionnaires and checklists focusing on skills, such as performance skills and general clinical skills [6]; however, the accuracy of self-assessment has been questioned [6,7]. Reviews have indicated that students are only moderately able to self-assess performance [8], and in comparing physician self-assessment to observed assessment, a positive association was demonstrated in only 7 of 20 studies [6]. None of the studies used self-efficacy as a method for self-assessment [6,8]. The authors recommend a more thorough understanding of the insights of physicians and their ability to reflect. Furthermore, they recommend that the research include the appraisal of perceived self-efficacy, which they describe as promising [6]. A recent study, in which a communication-training program was assessed using video ratings and self-assessment of self-efficacy, found that the improvement in self-efficacy was greater than the improvement based on the video ratings; however, different scales were used and no direct comparison between the two evaluation methods was performed [9].

Self-efficacy is widely used for self-assessing the outcome of communication skills training [10-16], and compared with other self-assessment tools, self-efficacy is not merely a passive reflection of performance, but has also shown to be a part of a self-fulfilling prophecy that affects performance [7]. Self-efficacy is a key element of social cognitive theory and refers to a person’s estimate of her or his ability to perform a specific task successfully [17]. The theory provides a framework for understanding how a person’s self-efficacy may affect the person’s behaviour [17,18], and research has shown that self-efficacy plays a predictive and mediating role in relation to motivation, learning, and performance [18,19].

Personal experiences, such as participating in role playing, is the most powerful source of creating a strong sense of efficacy, and educational programmes based on social cognitive theory and programmes using role playing have proved to be particular successful when evaluated on the self-efficacy scale [19]. Nearly all studies describing communication skills training include role playing [5]; however, neither the reliability of perceived self-efficacy nor the correlation between self-efficacy scores and more objective scores are known.

Thus, the aim of this study was to determine how medical students’ perceived self-efficacy of specific communication skills is compared to the evaluation of simulated patients and observers.

## Method

### Participants and design

All of the medical students who were signed up for an Objective Structured Clinical Examination (OSCE) at the medical school of the University of Southern Denmark in November 2010 were included.

All of the medical students had completed specific communication training consisting of 11 ECTS-points during the bachelor’s program. The students were in the 7th semester of the medical school and had completed a course on communication (9,5 ECTS) and a clinical residency focusing on communication training (15 ECTS-points).

As a part of the OSCE, the students were presented with paediatric and obstetric cases at 10 written and oral stations. For the purpose of comparison of the medical students’ perceived self-efficacy and the evaluation of the observers and patients, one oral case at one station was selected. A simulated patient and an observer evaluated the student performances in the “patient-physician interaction” at this OSCE station immediately after the performance.

The simulated patients were trained actresses. The observers were doctoral (Ph.D.) students affiliated with the research unit of general practice, primarily being medical doctors with clinical experience before entering the Ph.D. course of study.

After the examination the students were asked to assess their self-efficacy according to specific communication skills.

### Questionnaires

The Calgary-Cambridge Observation Guide Checklist [4] formed the basis for the outcome measures used in the questionnaires to the students, the simulated patients, and the observers. Twelve items were chosen, covering all 6 domains of the checklist (initiating the session, gathering information, building relationship, giving information, explaining and planning, and closing the session).

The students were asked to assess how confident they felt being able to successfully manage each of the 12 different communication skills rated on a Likert scale in categories 1–5 (strongly disagree to strongly agree). The simulated patients and the observers were asked to assess how the students succeeded in managing the 12 skills rated on a similar Likert scale.

### Validation of the questionnaires

A pilot test was performed to assess the feasibility of answering the questionnaires for the standardised patients and the observers within the time available during the exam, to test the inter-rater reliability, and to assess the face and content validity for all of the questionnaires. The pilot testing took place during a similar OSCE examination 6 months prior to the study. A total of 37 students, 7 standardised patients, and 2 pairs of observers participated with 1 pair at each station.

In the pilot study, no differences in the responses of the observers existed for any of the two pairs.

Based on the feedback from the participants, a detailed instruction to ensure greater consistency in the use of the scale and to improve the information to the simulated patients about the questionnaire was included; thus, the participants should only evaluate the communication of the students and not their medical competencies. Furthermore, one question was rephrased, and one of the questions was deleted because two of the other questions overlapped.

### Statistical analysis

We used an extended Rasch model [20,21] for the analysis of the data. This model tacitly assumes that differences in scores between items do not depend on the raters within a given group (students, observers, and patients). And likewise it also assumes that differences in scores between raters, within a given group, are independent of the items. The extension of the basic Rasch models [22] allows for comparison of groups rating the same performances. Utilization of all grades of the Likert scale requires that the same range of scores is used for all items, and similarly for all raters. As this was not the case, the responses were dichotomized. It was determined that a cut-point between categories 3 and 4 showed the overall best performance in terms of numerical stability of the estimation, and hence served best for the comparisons of the rater groups. The extended Rasch model allows for assessing the effect of covariates, such as gender or age. This was however not possible in the present case due to too few observations within each category. Analysis was conducted using R-package eRm [21,23].

### Ethical considerations

The students participating in the study received verbal and written information about the aim of the study, the right to withdraw and the guarantee of confidentiality of the information given to the researcher.

According to Danish law, the study did not require approval from the Danish Scientific Ethical Committee as it was a non-intervention study only using questionnaires.

## Results

Eighty-four students participated in the examination, of whom 73 (87%) responded to the questionnaire, of these 42 were women, 29 men and 2 did not indicate sex. The response rate for the simulated patients and the observers was 100%.

### Comparison of student self-efficacy and observer and simulated patient evaluation scores

When including all 12 items evaluated in the dichotomized analysis (categories 4 and 5 versus 3 to 1), significantly more items were scored in categories 4 and 5 by the observers than by the students (−0.23; SE:0.112; p=0.002).

When analysing the items individually, however, a statistically significant difference only existed for the following items: ‘Structure interview in logical sequence’ (item 3); and ‘Attend to time keeping, and keeping interview on task’ (item 4; Table 1).

**Table 1:** Comparison of students’ and observers’ and students’ and simulated patients’ assessment of selected items from the Calgary Cambrigde Guide checklist

	**Students vs. Observers**			**Students vs. Patients**		
**Items**	**Students N=73%**	**Observers N=84%**	Estimate* (0.95% CI)	**Students N=73%**	**Patients N=84%**	Estimate* (0.95% CI)
1: Identify problems the patient’s wishes to address	61.4	50.0	1.29 (0.64; 2.59)	61.4	68.4	2.25 (0.98; 5.18)
2: Use concise, easily understood, jargon free language	64.3	78.9	0.47 (0.21; 1.05)	64.3	64.9	1.13 (0.57; 2.21)
3: Structure interview in logical sequence	28.1	52.6	0.44 (0.22; 0.89)*	28.1	50.9	3.00 (1.28; 7.06)*
4: Attend to time keeping, and keeping interview on task	33.3	59.6	0.25 (0.10; 0.61)*	33.3	56.1	3.00 (1.28; 7.06)*
5: Use appropriate non-verbal behaviour	78.9	80.7	1.13 (0.43; 2.92)	78.9	77.2	0.79 (0.36; 1.73)
6: Provide support: express concern, understanding, and willingness to help	82.5	77.2	1.83 (0.68; 4.96)	82.5	71.9	0.59 (0.27; 1.28)
7: Share thought and reflection with the patient	52.6	59.6	0.63 (0.28; 1.38)	52.6	54.4	1.07 (0.53; 2.16)
8: Clarify patient’s prior knowledge and wish for information	50.9	59.6	0.80 (0.37; 1.71)	50.9	52.6	0.94 (0.46; 1.90)
9: Check patient’s understanding	49.1	32.1	2.25 (0.98; 5.18)	49.1	47.4	0.81 (0.39; 1.69)
10: Negotiate mutual plan of action	45.6	47.4	0.64 (0.28; 1.49)	45.6	45.6	0.94 (0.46; 1.90)
11: Contract with patient the next steps for patient and physician	61.4	67.9	0.82 (0.41; 1.67)	61.4	57.9	0.87 (0.43; 1.79)
12: Summarise session briefly and clarify plan of care	43.9	42.9	1.14 (0.56; 2.34)	43.9	50.9	1.86 (0.74; 4.66)

* p<0.05%.

Estimates and 95% CI for the odds of the response pattern ratio (0,1)/(1,0). The Proportion of responders in category 4–5 on a scale from 1–5 (strongly disagree to strongly agree) according to each item.

When comparing the student and simulated patient scores, significantly more items were scored in categories 4 and 5 by the patients than the students (0.177; SE:0.109; p=0.037).

Looking at the questions individually, showed that as for the comparison between the students and the observers only for items 3 and 4 the differences between the proportion of students and patients were statistically significant (Table 1).

Based on the dichotomized scores, detection of differences between raters depended on the cut-point. Therefore, a cut-point between 4 and 5 discriminated more differences between the raters, but showed unstable estimation due to the sparse number of observations with a score of 5.

### Distribution of student self-efficacy score, and observer and patient assessment scores

**Figure 1:**
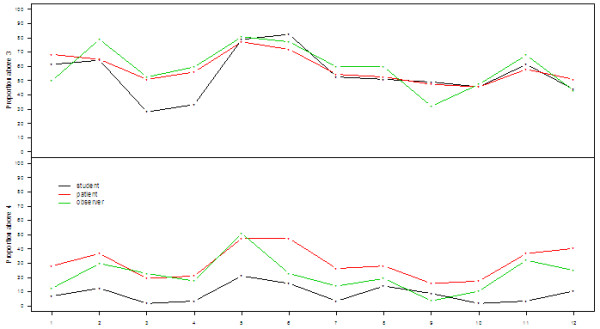
Proportions of responders with answers in category 4 or 5 (top panel) and in category 5 (lower panel) for students’, observers’ and patients’ assessment of selected items from the Calgary Cambrigde Guide with answers in category 4–5 on a scale from 1–5 (strongly disagree to strongly agree).

Figure 1 shows that for a cut-point between 3 and 4 (top panel), the proportion of students with a self-efficacy score of 4 or 5 approached the proportion of patients and observers giving a score of 4 or 5 for the performance. For items 3 and 4, the proportion of students giving scores > 3 was lower than that of observers and patients. For item 9 (Check patient’s understanding), the proportion of observers giving a score > 3 was slightly lower than that of the students and patients.

The lower panel in Figure 1 shows the proportion of observers and patients scoring 5 for the items assessed. The shapes of the curves are very similar to that of the students, though at a considerably higher level. The observers only scored the performance for item 9 lower than the students themselves. The figure thus illustrates that the students generally have a self-efficacy score, which is lower than that of the patients and observers.

## Discussion

The current study showed that students scored their communication skills lower compared to observers or simulated patients. The differences were, however, driven by only 2 of 12 items. The results indicate that by using self-efficacy scores, the students do not overestimate their skills, while such was the case in studies in which a self-assessment tool was used in comparison with external scores [24], and in other OSCE scenarios in which the communication of the students was scored by simulated patients [8].

The fact that the shapes of the three curves were very similar indicates a high agreement between the evaluation of the performance of different items by the students, patients, and observers.

Nevertheless, some differences were shown. As compared to the other items, the fewest number of students gave a high self-efficacy score for item 3 (‘Structure interview in logical sequence’) and item 4 (‘Attend to time keeping, and keeping interview on task’), indicating a relatively low self-efficacy for these tasks as compared with self-efficacy in general. Both patients and observers rated these items significantly higher than the students, indicating different expectations according to these skills or the fact that these items functioned most poorly as self-assessment items. In contrast to the other items, items 3 and 4 pertained to the overall structure of the communication, while the other items focused on specific communication skills (e.g., identify problems, use appropriate language, and check patient understanding), and therefore were easier to assess.

The statistical analyses were based on a cut-point between 3 and 4. Changing the cut-point to between 4 and 5 showed the same tendency, but caused unstable estimations and increased uncertainty due to the small number of observations. However, the distance between the curves increased, indicating that the students generally scored themselves lower than the observers and simulated patients. By using the Calgary Cambridge Observation Guide as a basis for the self-efficacy and objective assessment scores, the evaluation tools closely match the communication skills taught, and thus give a clear sense of the impact of the training on the different items assessed [5]. In a study in which communication and clinical skills were assessed using seven different instruments, the Calgary Cambridge scale was the most powerful measure for discriminating skilful from less skilful communicators [25].

Among the main limitations of the study is the lack of a gold standard, which is often a fundamental problem in these types of studies. In the OSCE examination, the assessment of the observers is considered the gold standard; however, the validity of this “true value” has been questioned in a study showing a large variability in examiner scores due to examiner stringency behaviour [26]. In our pilot study, we demonstrated high agreement between the two observers, supporting the validity of the scores of the observers.

Another limitation of the study was that the comparison was based on different types of questions. The observers and patients were asked to assess the skills of the students, while the students were not asked to evaluate their performance, but were asked to assess how confident they felt being able to successfully manage the tasks.

As a consequence, we did not expect to achieve exact agreement between the ratings of the observers and students. However, the obvious strength of using self-efficacy measures instead of self-assessment measures is that the self-efficacy scores mirror not only the actual performance, but also the perceptions of the students regarding the specific competencies in general. Furthermore, a large amount of research demonstrates that self-efficacy appears to be a significant factor in learning because it affects the motivation, exertion, learning, and performance of the students [19].

No published studies have investigated self-assessment compared with observed methods using self-efficacy measures. The results in the current study indicate that self-efficacy based on the Calgary Cambridge scale is a reliable tool. Although more research is required to confirm our findings, we suggest that the self-efficacy scale may be used to assess the outcome of training in which objective ratings are not an option (e.g., as an evaluation tool used for the purpose of assessing the outcome of continuous professional development). As self-efficacy obviously is a self-evaluation it can not be used as a tool for summative assessments for testing the individual students. However, the results show that it can be a useful and reliable tool for formative assessments and thus can be used to make decisions about teaching and instruction situations by identifying the strengths and weaknesses in the skills of the health professionals and thereby also for identifying the need for education and training as a basis for continuous development.

## Conclusion

This study showed that students scored their communication skills lower compared to observers or simulated patients. The differences were driven by only 2 of 12 items.

The results in this study indicate that self-efficacy based on the Calgary Cambridge Observation guide seems to be a reliable tool that can be used for formative assessment of health professionals.

## Competing interest

The authors declare that they have no competing interests.

## Authors’ contribution

JA, PEK, JLT, DEJ, ACHØ and PEK designed the study. JLT, DEJ and ACHØ collected the data. JA wrote the first draft of the paper. RH performed the statistical analyses. All authors participated in the interpretation of the data, they contributed to the critical revision of the paper and approved the final version of the manuscript.

## Pre-publication history

The pre-publication history for this paper can be accessed here:

http://www.biomedcentral.com/1472-6920/13/49/prepub
